# Energy Landscapes for the Unitary Coupled Cluster Ansatz

**DOI:** 10.1021/acs.jctc.4c01667

**Published:** 2025-02-16

**Authors:** Choy Boy, Maria-Andreea Filip, David J. Wales

**Affiliations:** Yusuf Hamied Department of Chemistry, University of Cambridge, Cambridge CB2 1EW, U.K.

## Abstract

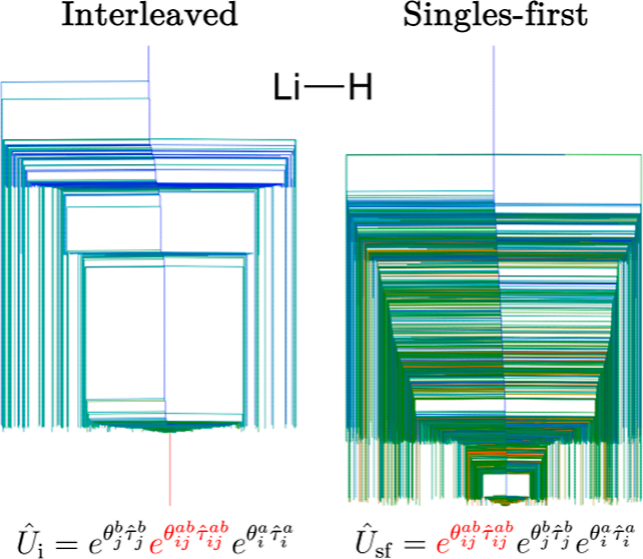

The unitary coupled cluster (UCC) approach has been one of the most popular wavefunction parametrizations for the variational quantum eigensolver due to the relative ease of optimization compared to hardware-efficient ansätze. In this contribution, we explore the energy landscape of the unitary coupled cluster singles and doubles (UCCSD) wavefunction for two commonly employed benchmark systems, lithium hydride and the nitrogen dimer. We investigate the organization of the solution space in terms of local minima and show how it changes as the number and order of operators of the UCC ansatz are varied. Surprisingly, we find that in all cases, the UCCSD energy has numerous low-lying minima connected by high energy transition states. Additionally, the energy spread of the minima that lie in the same band as the global minimum may exceed chemical accuracy, making the location of the true global minimum especially challenging.

## 1 Introduction

The advent of quantum computing as a viable computational model has led to the development of novel methods and algorithms designed for this architecture. Quantum chemistry has been a popular target for these algorithms due to the intuitive mapping between the electronic structure problem and qubit systems as well as the potential utility of even moderately sized quantum processors.

In the current era of noisy intermediate-scale quantum devices, hybrid quantum-classical algorithms have been an important focus for research. Many of these approaches are variational algorithms, in which some cost function is computed on the quantum device and the corresponding parameters are optimized on a classical computer. In the context of quantum chemistry, the variational quantum eigensolver^1^ (VQE) is perhaps the most widely used. In this approach, one attempts to minimize the energy of some parametrized wavefunction.

The quality of VQE energies and wavefunctions is strongly dependent on the choice of parametrization. Many conventional functional forms employed in quantum chemistry, such as configuration interaction (CI) and coupled cluster (CC), violate the unitarity constraints imposed by the nature of the quantum architecture. However, there exist unitary formulations, such as unitary coupled cluster^2−5^ (UCC), which, while unwieldy in a classical setting, have been extensively used for VQE. Quantum architecture inspired parametrizations have also been devised, such as the hardware efficient ansatz^6^ (HEA), which aim to optimize the use of quantum hardware resources by maximizing quantum gate economy via reducing circuit depth. Although such an ansatz is significantly more compact than UCC, multiple layers may be needed to reach suitable accuracy. As the number of layers increases, optimization is found to be challenging due to the occurrence of areas of configuration space with relatively low gradients, known as barren plateaux.^7^ Finally, there exist a range of alternative approaches, which combine physical insight with depth reduction schemes.^8−16^ These methods include qubit coupled-cluster approaches,^8,11,13^ which avoid some of the complexity of encoding Fermionic excitation operators for a quantum computer, adaptive methods,^9,13^ and symmetry-preserving ansätze.^10,12,16^ Because of the relatively straightforward optimization relative to HEAs, the UCC form of the wavefunction remains the basis for many of these algorithms,^8,9,11,13,15^ which generally aim to reduce the complexity.

In this paper, we investigate the energy landscapes of UCC ansätze in two archetypal molecules for quantum chemistry benchmarking, namely, lithium hydride and the nitrogen molecule. Here, the energy landscape is defined in terms of local minima and the transition states that connect them. Our contribution builds upon previous studies of variational quantum algorithms (VQAs) including the hardware-efficient^17^ and quantum approximate optimization algorithm (QAOA)^18^ ansätze. We consider both complete UCC singles and doubles wavefunctions, as well as truncations of them, obtained from minimal quantum Monte Carlo (QMC) calculations.^19^ We construct the landscape from local minima obtained by basin-hopping global optimization,^20−22^ connected by transition states obtained using the doubly-nudged^23,24^ elastic band^25−28^ (DNEB) algorithm to propose candidate configurations and refining them by hybrid eigenvector-following.^29−31^ We explore the organization and properties of these landscapes, as well as the effects of parameter number and operator ordering. The paper is structured as follows: Section 2 gives an overview of the theory behind VQE and the UCC ansatz as well as the techniques used to construct and assess the energy landscapes. Results and discussion are given in Section 3, followed by our conclusions in Section 4.

## 2 Theory

### 2.1 Variational Quantum Eigensolver

Consider some parametrized wavefunction
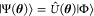
1where *Û*(**θ**) is a parametrized unitary operator and |Φ⟩ is a simple initial state, often taken to be the Hartree–Fock (HF) state in Fermionic systems. This wavefunction can be optimized by employing the variational principle, with global optimization of the expectation value of the energy generating the best possible approximation to the ground-state wavefunction of the given parametric form. However, the solution landscape may support numerous local minima, as we shall see.

In VQE, for a given set of parameters **θ**, the expectation value of the energy, and potentially the gradient, is computed on a quantum device where this operation can be carried out efficiently. These data are then passed to an optimization routine running on a classical computer, which updates the parameters. This cycle continues until specified convergence criteria are met.

### 2.2 Unitary Coupled Cluster Approach

In this paper, we focus on the UCC ansatz,^2−5^ in which *Û*(**θ**) is given as an exponential
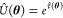
2where
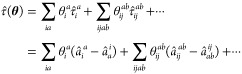
3and  is a Fermionic operator corresponding to excitation of electrons from orbitals *p*, *q*, ... to orbitals *r*, *s*, ... In eq 3, the indices *i*, *j* (*a*, *b*) run over orbitals occupied (unoccupied) in the HF state. While in principle eq 3 runs up to *N*-fold excitations, where *N* is the number of electrons in the system, it is usually truncated after the double excitation term, leading to the unitary coupled cluster singles and doubles (UCCSD) ansatz. Using this ansatz, the variational energy is given by
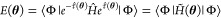
4

Due to the presence of so-called de-excitation operators (e.g., ) in , the Baker–Campbell–Hausdorff (BCH) expansion of the operator *H̃*(θ) is nonterminating, making the UCC method exponentially scaling in a classical setting, irrespective of the excitation truncation order. However, this issue does not pose a problem when the energy is measured by using a quantum device. To make this measurement possible, both  and *Ĥ* must be transformed into qubit operators. One way to do this is the Jordan–Wigner encoding,^32^ which is described in Appendix A.

Additionally, to make *Û*(θ) easier to represent in a quantum circuit, VQE applications commonly use the disentangled form of the UCC ansatz, which can be obtained by a first-order Trotter-Suzuki expansion
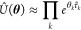
5where *k* represents some indexing of the terms in . The expressivity of this ansatz depends on the ordering chosen for this expansion.^33,34^ In this work, we will focus on the UCCSD truncation of the UCC ansatz, which is the most widely used in applications. We consider two possible orderings of the excitors: the singles-first order
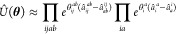
6and the interleaved order^33^
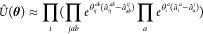
7For a given excitation operator , we employ the following formulation to apply the operator  to a wavefunction |Ψ⟩:^35^

**Case I**: If |Ψ⟩ = 0 and |Ψ⟩ ≠ 0, then  = cos(θ_*p*_)|Ψ⟩ + sin(θ_*p*_)|Ψ_*p*_⟩, where |Ψ_*p*_⟩ is the partial excited state after applying  to |Ψ⟩,

**Case II**: If |Ψ⟩ ≠ 0 and |Ψ⟩ = 0, then  = cos(θ_*p*_)|Ψ⟩ − sin(θ_*p*_)|Ψ^*p*^⟩, where |Ψ^*p*^⟩ is the partial deexcited state after applying  to |Ψ⟩,

**Case III**: If  and , then .

Since the number of states in the computational basis increases exponentially with *N*, and the vast majority of transformations belong to **Case III**, we dynamically vary the size of the array encoding |Ψ⟩ in our statevector simulation based on the number of constituent states with nonzero coefficients at every sequential application of , thus greatly reducing the computational cost of classically evolving |Ψ⟩.

### 2.3 Quantum Monte Carlo

In this work, we investigate how the landscape of the UCCSD ansatz changes with the parameters. Given a complete UCCSD ansatz for a system, we are interested in obtaining useful truncations. For this purpose we use the unitary coupled cluster Monte Carlo (UCCMC) approach,^35^ which has been shown to generate good truncations of UCC ansätze.^19^

In this approach, the parameters of the UCCSD wavefunction are obtained, not by variational optimization, but by stochastic imaginary-time evolution, using a projector Monte Carlo approach.^35−41^ These methods start from the imaginary-time Schrödinger equation
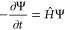
8where *t* is imaginary time. For a time-independent Hamiltonian, eq 8 has a solution of the form
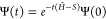
9where *S* is an integration constant, Ψ(0) is the initial wavefunction, and Ψ(*t*) is the wavefunction at imaginary time *t*. If the initial overlap with the true ground-state Ψ_FCI_ is nonzero, then in the infinite imaginary time limit, this approach recovers the ground state of the system
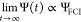
10Most Hilbert space QMC approaches are obtained by splitting the imaginary time propagator  into *n* steps and taking a first-order Taylor expansion. In that case
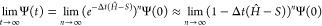
11This formulation can be recast as an iterative equation
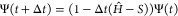
12For a parametrized wavefunction Ψ(**θ**) for which there exists a set of Slater determinants Φ_*p*_ such that for every parameter θ_*p*_, , update equations for each parameter can be obtained by projecting eq 12 onto different determinants. Various popular quantum chemistry ansätze fall into this category, including CI,^38^ CC,^40^ and UCC.^35^ The parameter update equations are then given by
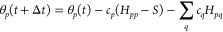
13where *c*_*p*_ is the corresponding coefficient of determinant Φ_*p*_ in the CI expansion of Ψ(*t*) and .

The above equation can be interpreted as the population dynamics of a set of signed particles existing in the Hilbert space with the population of each determinant proportional to θ_*p*_. This equation can be propagated stochastically, by the following processes:^38^

- *Spawning* of particles from determinant Φ_*q*_ to Φ_*p*_ occurs with probability proportional to *c*_*q*_*H*_*pq*_ and opposite sign.
- *Death/cloning* of particles on determinant Φ_*p*_ occurs with probability proportional to *c*_*p*_(*H*_*pp*_ – *S*) and opposite sign.
- *Annihilation* cancels out particles of opposite signs on each determinant.

By varying the parameter *S* to reach a steady-state population, a stochastic approximation of the true ground state can be reached, with both *S* and
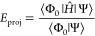
14as valid estimators for the corresponding energy.

QMC approaches, such as UCCMC, have been shown to identify important contributions to the wavefunction early in the propagation and as such may be used as a means of selecting important parameters without the need to converge the calculation.^42^

### 2.4 Basin-Hopping Global Optimization

To explore the energy landscapes of UCC ansätze, basin-hopping global optimization^20−22^ can be performed to obtain samples of local minima for the construction of kinetic transition networks.^43−45^ This approach typically involves combining the optimization routine within the VQE algorithm with a Metropolis accept/reject criterion:^46^ a given basin-hopping step *k* with corresponding energy *E*(**θ**_*k*_) is accepted if either *E*(**θ**_*k*_) < *E*(**θ**_*k*–1_), or with probability equal to exp[−(*E*(**θ**_*k*_) – *E*(**θ**_*k*–1_)/*k*_*B*_*T*)] if *E*(**θ**_*k*_) ≥ *E*(**θ**_*k*–1_), where *T* is the basin-hopping temperature for a given regime. If *E*(**θ**_*k*_) is not accepted, a random perturbation will be applied to the previous coordinates **θ**_*k*–1_, and the accept/reject sequence proceeds until a suitable convergence criterion is attained. In this study, we utilize the limited-memory Broyden–Fletcher–Goldfarb–Shanno (L-BFGS)^47−51^ procedure as the minimizer for each basin-hopping quench, which requires the evaluation of the gradients associated with each operator in , where *Q* is the number of UCC operators for a given system. The quantum circuits of UCC ansätze can typically be systematically decomposed as a sequence of one- and two-qubit gates, which allow us to use of the parameter-shift rule to calculate the gradients of the parametrized one-qubit rotation gates.^52^ However, the exponential rise in the number of quantum gates as the molecular complexity grows^8^ greatly increases the computational cost of evolving the circuit with different parametrized angles for each rotation gate in order to find the gradients. Instead, in a classical simulation, the final wavefunction |Ψ(**θ**)⟩ can be evolved in a backward schedule to recursively obtain the analytic gradients for operator number *Q*, *Q* – 1, ..., 1 in that order.^53^ The analytic form of the UCC gradient is
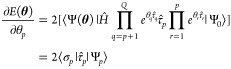
15using the notation  and . Algorithm 1 outlines the general procedure for calculating the analytic UCC gradients, decreasing the time complexity from *O*(*Q*^2^) to *O*(*Q*).^53^ It is also essential to continuously employ the dynamic-array technique during the evaluation of |Ψ_*p*_⟩ and |σ_*p*_⟩ since the number of ket states with nonzero coefficients may increase due to the generation of new states from applying the UCC operators sequentially in the reverse order.
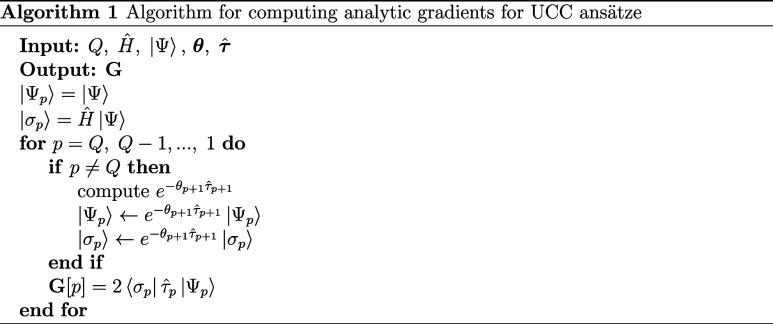


Based on the initial collection of minima obtained from basin-hopping global optimization, kinetic transition networks can be constructed by identifying transition state candidates between pairs of minima using the doubly-nudged^23,24^ elastic band^25−28^ (DNEB) algorithm, followed by accurate convergence using hybrid eigenvalue-following methods.^29−31^ The overlap matrix between two stationary points with wavefunctions |Ψ(**θ**_μ_)⟩ and |Ψ(**θ**_ν_)⟩, respectively, required to carry out discrete path sampling (DPS)^54,55^ for this analysis, *S*_μν_, is
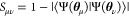
16The DPS procedure may also introduce new connected minima into the existing database. Thus, it is important to distinguish true minima with no negative eigenvalues in their respective Hessian matrix **H**_**θ**_ from higher-order saddle points, producing a sampled set of true minima of size *M*. By partial differentiating eq 15, the Hessian matrix element with parameter index (*i*, *j*), *h*_*ij*_, can be derived as
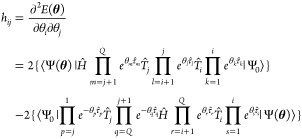
17We thus implement algorithm 2 for the efficient evaluation of the Hessian matrix for the set of stationary points obtained from the analysis of kinetic transition networks, allowing for a reduction in time complexity from *O*(*Q*^4^) to *O*(*Q*^2^). Since *h*_*ij*_ is composed of simple trigonometric functions with addition and multiplication operations, by Schwarz’s theorem **H**_**θ**_ must necessarily be symmetric, i.e. *h*_*ij*_ = *h*_*ji*_, so one need only consider Hessian matrix elements for *i* ≥ *j*, further reducing the number of calculations by a factor of .
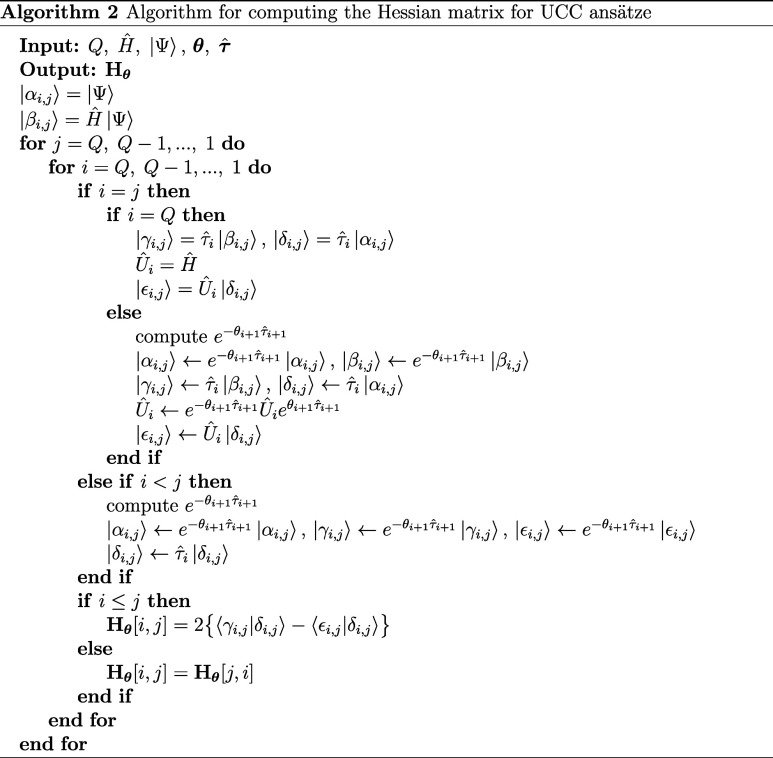


## 3 Results

### 3.1 Methodology

We investigate the energy landscapes of the UCCSD wavefunctions for the LiH and N_2_ molecules using STO-3G basis sets,^56,57^ near the corresponding equilibrium geometries. For LiH, we carry out all-electron calculations, while for N_2_, the 1*s* core is frozen. These systems then correspond to 12 and 16 qubit systems, respectively.

We begin by running short UCCMC calculations using the interleaved parameter order to obtain initial approximations of the UCC amplitudes. For LiH, we performed 50,000 Monte Carlo iterations, with an imaginary time step *dt* = 0.005 and a target population of 5000 walkers. For N_2_, we performed 3000 steps, with the same time step and a target population of 10,000 walkers. Both of these calculations took less than a minute on a six-core desktop computer. We then select all of the amplitudes above a certain amplitude threshold to include in a simplified UCCSD ansatz. We exploit the full point group symmetry preserving UCCSD ansätze and corresponding truncations, including only amplitudes greater than a given threshold. For LiH, this setup leads to ansätze with 34 (|θ| > 0) and 20 (|θ| > 0.001) parameters. For N_2_, we consider ansätze with 53 (|θ| > 0), 38 (|θ| > 0.01) and 17 parameters (|θ| > 0.05).

The visualization of the topological features associated with the energy landscapes of UCC ansätze can be carried out by plotting disconnectivity graphs,^58,59^ where at regular energy thresholds *E* with energy interval Δ*E*, superbasins are constructed from groups of minima that are connected via transition states below the threshold *E*. As discussed in the following section, in some cases the minima close in energy with respect to the global minimum possess rotation amplitudes close to {0, ±π}, whereas for higher energy minima, some amplitudes have values close to . When the corresponding (de)excitation operators are applied, parameters exactly equal to {0, ±π} would simply introduce a phase in the wavefunction, while those equal to  correspond to a complete rotation between two determinants, as can be seen from **CASE I–III**. We therefore introduce the metric *D* as a means of visualizing the extent of state swapping for each minimum based on their rotation amplitudes
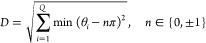
18The *D* values of each minimum are used to color the disconnectivity graphs through a continuous spectrum, with the lowest and highest *D* values assigned to red and blue, respectively. As outlined in subsequent sections, unlike molecular systems, such as native proteins that can reach the global minimum via relaxation on a well-funnelled energy landscape,^60−64^ it was found that the energy landscapes of UCC ansätze resemble more closely those of glassy systems.^65−69^ Here, minima situated in discrete energy neighborhoods are separated by comparatively high energy barriers. With this consideration in mind, the disconnectivity graphs featured in this work were drawn so that individual minima within a range of 10^–5^ Ha were merged, allowing the visualization of the energy neighborhoods and the barriers between them with greater clarity. We also investigated these energy landscapes from a thermodynamic perspective using an analogue of the heat capacity *C*_*v*_(*T*) for various UCC ansätze over a specified temperature range. *C*_*v*_(*T*) is calculated from the total partition function *Z*(*T*) as
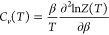
19where β = 1/*k*_*B*_*T*. In the superposition approach,^70−74^*Z*(*T*) is written in terms of the partition functions *Z*_*m*_(*T*) for all the *M* local minima
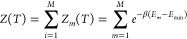
20Here, we have neglected the configurational density of states associated with the basin of attraction of each minimum; for a molecular partition function, this approximation is equivalent to assuming that all the normal mode vibrational frequencies are identical for every isomer. The resulting heat capacity curves typically exhibit a main larger peak and several smaller peaks situated at lower temperature. As the integrated area enclosed under the heat capacity curve corresponds to the increase in the internal energy between different phase-like forms, the height of the main peak provides insight into the primary transition from the lowest energy neighborhood containing the global minimum to the higher energy neighborhoods, while the smaller peaks describe secondary transitions within narrower energy ranges.

Initial Hartree–Fock energies and orbital integrals were obtained from PySCF.^75^ UCCMC calculations were carried out using the HANDE-QMC package.^76^ Basin-hopping global optimization was performed using the GMIN^77^ program, and DPS to construct the connected networks of minima employed the OPTIM^78^ and PATHSAMPLE packages.^79^ The disconnectivity graphs were calculated with the disconnection DPS program.^80^

### 3.2 Energy Landscapes of LiH

We begin by considering the energy landscape of the UCCSD ansatz for LiH with a bond length of *r* = 1.6 Å. The full UCCSD ansatz for this system consists of 34 operators, of which eight are single-excitation operators and 26 are double-excitation operators. Limiting the ansatz to those operators that are identified as having amplitudes greater than 10^–3^ by a short UCCMC simulation leaves 20 operators, of which four are single-excitation and 16 are double-excitation operators.

**Figure 1:**
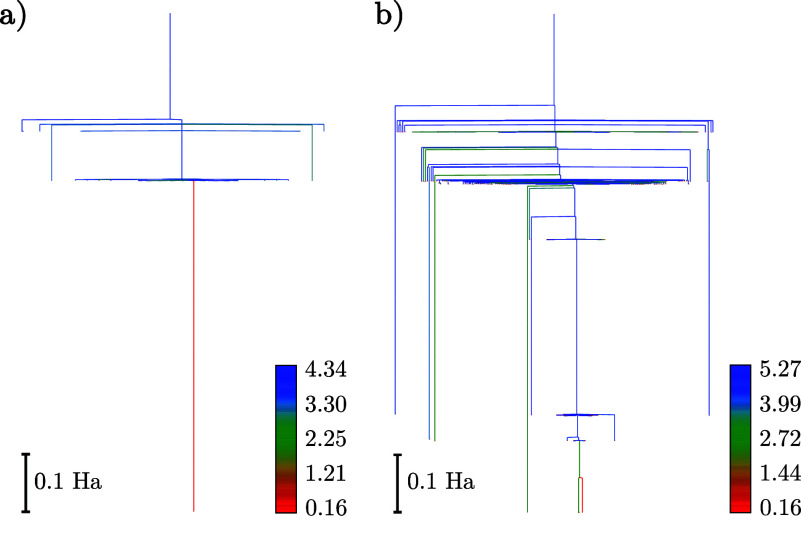
Disconnectivity graphs of the 20-operator LiH UCCSD ansatz with *r*_Li–H_ = 1.6 Å for the interleaved (a) and singles-first (b) cases, respectively. Individual minima were merged if they are within 10^–5^ Ha and are colored by their respective *D* values.

**Figure 2:**
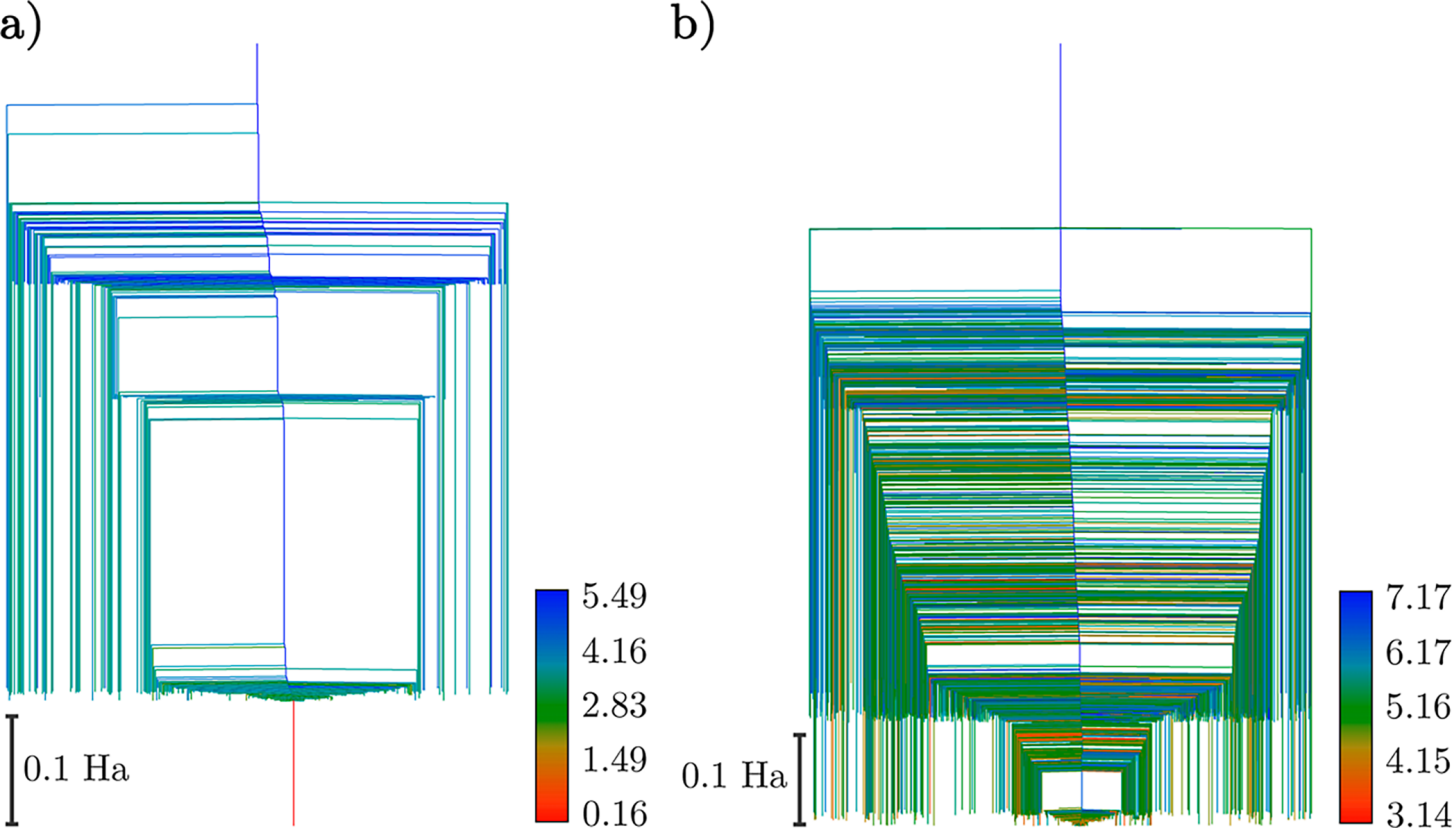
Disconnectivity graphs of the 34-operator LiH UCCSD ansatz with *r*_Li–H_ = 1.6 Å for the interleaved (a) and singles-first (b) cases, respectively. Individual minima were merged if they lie within a range of 10^–5^ Ha and are colored by their respective *D* values.

**Figure 3:**
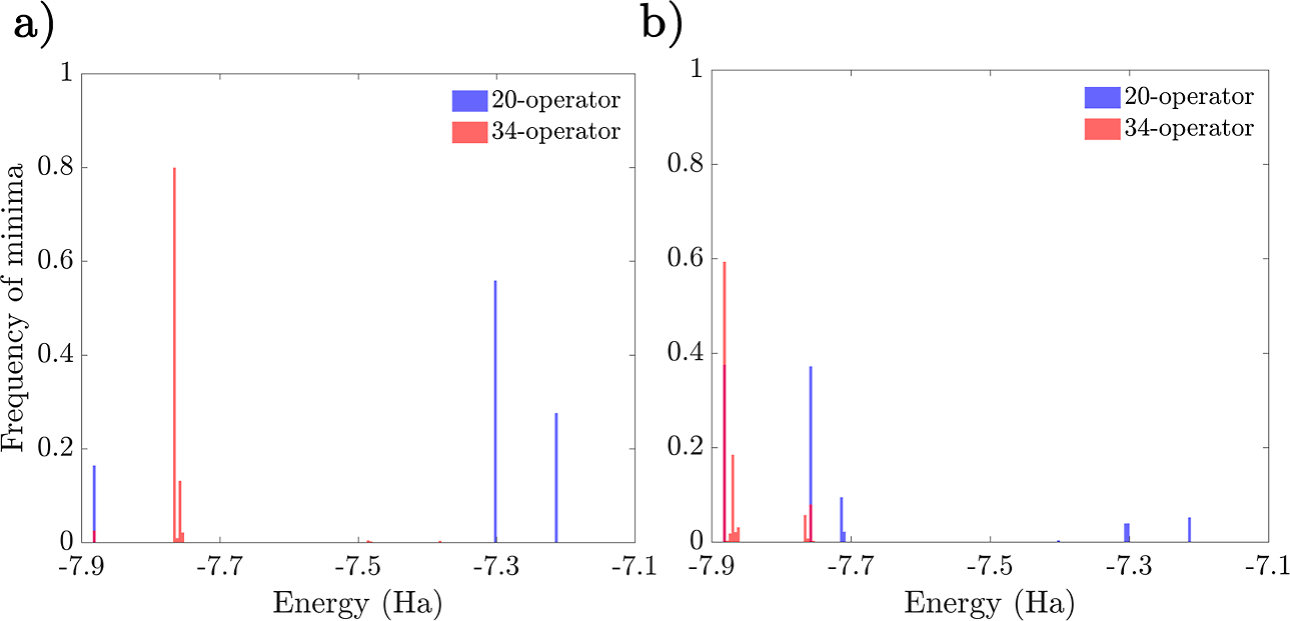
Minima histogram distributions for the interleaved (a) and singles-first (b) LiH UCCSD ansatz with *r*_Li–H_ = 1.6 Å. The 20- and 34-operator cases are shown in blue and red, respectively.

**Figure 4:**
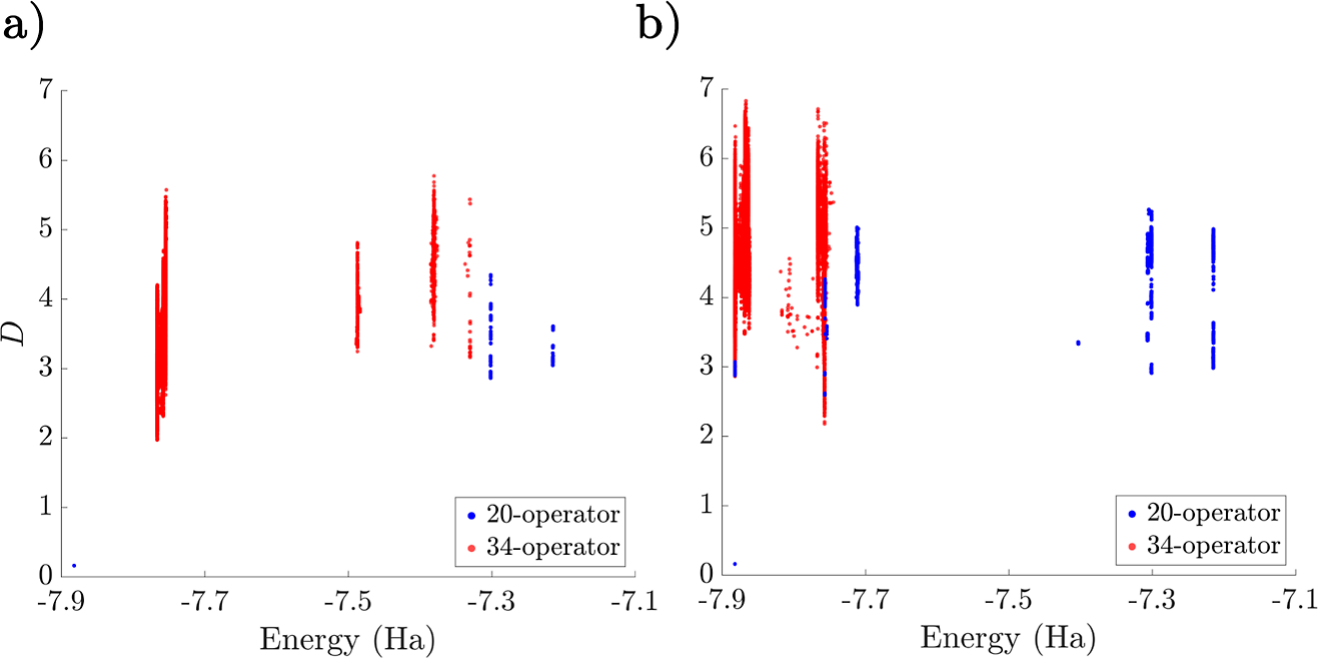
Scatter plots of the interleaved (a) and singles-first (b) LiH UCCSD ansatz for *r*_Li–H_ = 1.6 Å based on the corresponding total energy and *D* values of the minima. The 20- and 34-operator cases are shown in blue and red, respectively.

The disconnectivity graphs^58,59^ corresponding to the 20- and 34-parameter ansätze are shown in Figures 1 and 2, where the bottom of each line corresponds to the energy of a local minimum of the expectation value of the energy. These branches are connected at the lowest energy threshold, for which the minima can interconvert via pathways mediated by one or more transition states. The energy thresholds for this connection analysis are chosen at regular intervals to highlight the organization of the solution space. Lines are colored according to the *D* value of the minima (see eq 18), denoting the scale of the deviation of the corresponding parameters from {0, ±π}, values, which only induce a phase change to the wavefunction. The corresponding energy distributions for the local minima are shown in Figure 3. Using the interleaved order suggested by Evangelista et al.^33^ for the 20-parameter ansatz, up to a resolution of 10^–5^ Ha, there is a single merged state containing the global minimum, which deviates from the true ground state in this basis by 1.6 × 10^–5^ Ha in energy (see Table 1), with a number of high-energy minima, separated by significant transition barriers. There is good correlation between energy and *D* values, which can be seen in Figure 4, where we show the much smaller spread of parameter values for states near the global minimum compared to those higher in energy. Employing instead the singles-first ordering increases the complexity of the landscape, introducing a variety of additional minima, including low-energy high-*D* minima close to the global minimum (see Table 1). This ordering affects the distribution of minima in Figure 3, with a larger proportion of low-lying minima, the barriers between which remain non-negligible.

**Table 1:** Energies of the Lowest Minimum, the Number of Sampled Connected Minima *M*, and the Energy Difference within the Set of Lowest Minima Found with Different UCCSD Ansätze for the LiH Molecule with *r*_Li–H_ = 1.6 Å

parameters	order	*M*	Δ*E*_*l*_ (Ha)	global minimum (Ha)	FCI (Ha)
20	interleaved	304	2.4 × 10^–6^	–7.882308	–7.882324
20	singles-first	39,745	2.3 × 10^–4^	–7.882308	
34	interleaved	280,022	2.9 × 10^–6^	–7.882315	
34	singles-first	62,193	2.3 × 10^–3^	–7.882315	

Increasing the number of parameters to 34 leads to a much larger number of minima, although for the interleaved ansatz, there remains a relatively narrow band of low-energy minima that contains the global minimum. These solutions are again characterized by parameter values close to 0 or ±π and are closer to the FCI energy (see Table 1). Using the singles-first parameter order in this case produces a glassy structure in the low-energy region of the energy landscape, with a large number of minima close in energy but separated by high-energy transition states. Once again, the vast majority of the additional minima that result from increasing the number of parameters have significantly larger *D* values than the global minimum and associated low-lying solutions for the interleaved ansatz.

To understand the propensity of the singles-first ansatz to introduce new minima with low energies but high *D* values, we consider the structure of the two ansätze in more detail. We start from a simple example including two disjoint single excitations (labeled *i* → *a* and *j* → *b*) and the corresponding double excitation (*i*, *j* → *a*, *b*). In this case
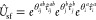
21and
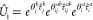
22are the singles-first and interleaved unitary operators, respectively.

**Figure 5:**
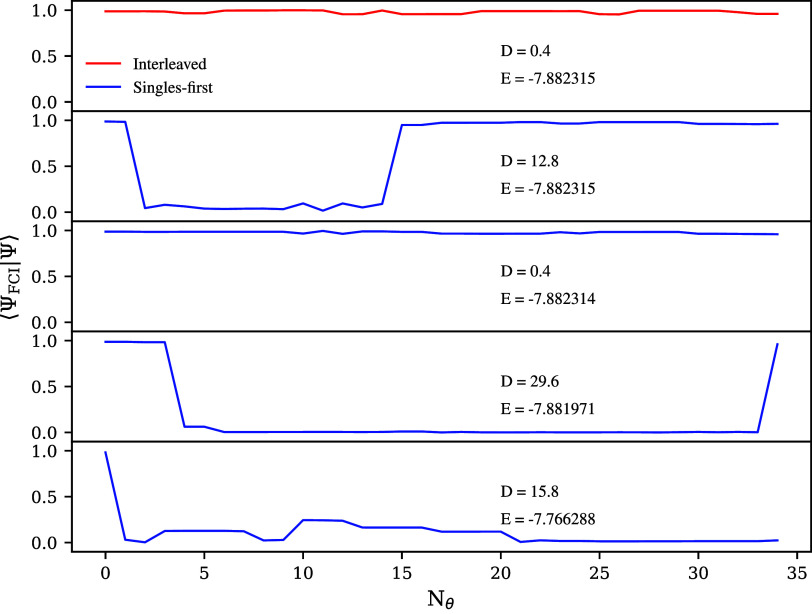
Example overlaps of selected local minima with the 34-parameter ansatz for LiH with the true ground-state FCI wavefunction as operators are applied sequentially. The top two panels show the lowest minima found for the interleaved and singles-first ansätze. The following three panels show representative minima with low energy and low *D*, low energy and high *D*, and high energy and high *D* values, respectively. Energies and *D* values for each minimum are noted in the respective panels. *N*_θ_ gives the number of excitation operators applied, with *N*_θ_ = 0 corresponding to the HF state.

In the singles-first case, if we start from the Hartree–Fock state and set all three parameters to θ_*i*_^*a*^ = θ_*j*_^*b*^ = θ_*ij*_^*ab*^ = ±π/2, then
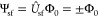
23In contrast, using the same parameters
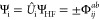
24because the central double excitation operator has no effect, falling into **CASE III**. In fact, it is part of the design principle of the interleaved ansatz that it is never able to “return” to a previous state.^33^ Therefore, if the initial state is a good approximation to the true ground state, the interleaved ansatz should only require relatively small changes to produce the best available approximation of the ground state as any large changes would be impossible to correct. In the singles-first ansatz, on the other hand, it is possible to choose parametrizations where applying the single excitation operators pushes the wavefunction very far from the ground state, but this issue is corrected by a double excitation operator later in the order. Such large changes correspond to large contributions to *D*, which can produce low energy minima with large *D* values. Examples of such minima are given in Figure 5, which tracks the overlap between the trial wavefunction and the true ground state as operators are sequentially applied.

In the global minimum of the interleaved case, the overlap remains consistently high. The same is true for the singles-first minimum shown in the middle panel, which has a comparable value of *D*. However, this is not the global minimum in the singles-first ansatz, which instead exhibits the behavior predicted above: the overlap drops sharply after the  excitation operator is applied and recovers after application of the  double excitation operator. There are various ways for this sort of behavior to be achieved, with another similar minimum shown in the fourth panel of Figure 5. The last panel shows instead a high energy minimum, where the overlap with the ground state remains negligible to the end of the propagation.

**Figure 6:**
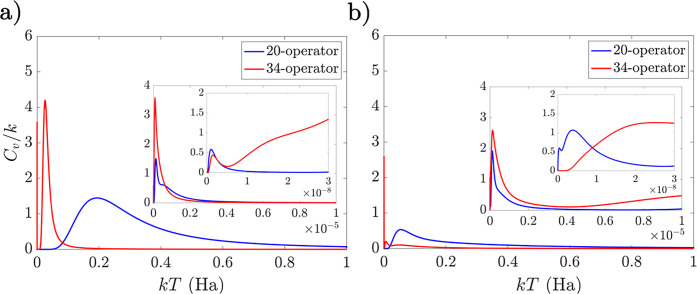
Heat capacity curves for the LiH UCCSD ansatz with *r*_Li–H_ = 1.6 Å for the interleaved (a) and singles-first (b) cases, respectively. The 20- and 34-operator cases for both operator orders are shown in blue and red, respectively.

Finally, analysis of the heat capacity curves of the LiH ansätze reveals a thermodynamic picture of how operator selection and order affect the distribution of minima within the UCC energy landscapes (Figure 6). Starting with a comparison of the number of operators, we found that the main heat capacity peak located above 0.01 Ha for the 34-operator case has a sharper profile than that for the 20-operator case with the interleaved ansätze. This result can be attributed to two main factors: as the 34-operator case has a larger proportion of minima situated in higher energy neighborhoods than that in the lowest energy neighborhood, the phase-like transition from the low-lying minima is sharper. However, due to smaller differences in energy between the lowest and higher minima neighborhoods, the change in internal energy for such a transition is lower, and thus, the area under the main peak is smaller. For the singles-first case, the main heat capacity peak of the full UCCSD ansatz also encloses a smaller area compared with the 20-operator case. However, as the proportion of minima in the lowest energy neighborhood is much greater, the main transition is not as large and the heat capacity peak is consequently not as sharp. The two insets of Figure 6 depict secondary, smaller features at lower temperatures produced by different bands of minima in the LiH ansätze series, which highlight the complex features of the energy landscape over a wide range of energy scales. For the 34-operator, singles-first case, the secondary peak at approximately 1.0 × 10^–6^ Ha is much sharper than the main peak due to the greater variation in the energies of the minima within the different bands. Thus, the heat capacity curves provide a visualization of the change in minima distributions between the 20- to the 34-operator ansätze, as well as the differences in the energy landscape profiles of both the interleaved and singles-first cases.

### 3.3 Energy Landscapes of N_2_

**Figure 7:**
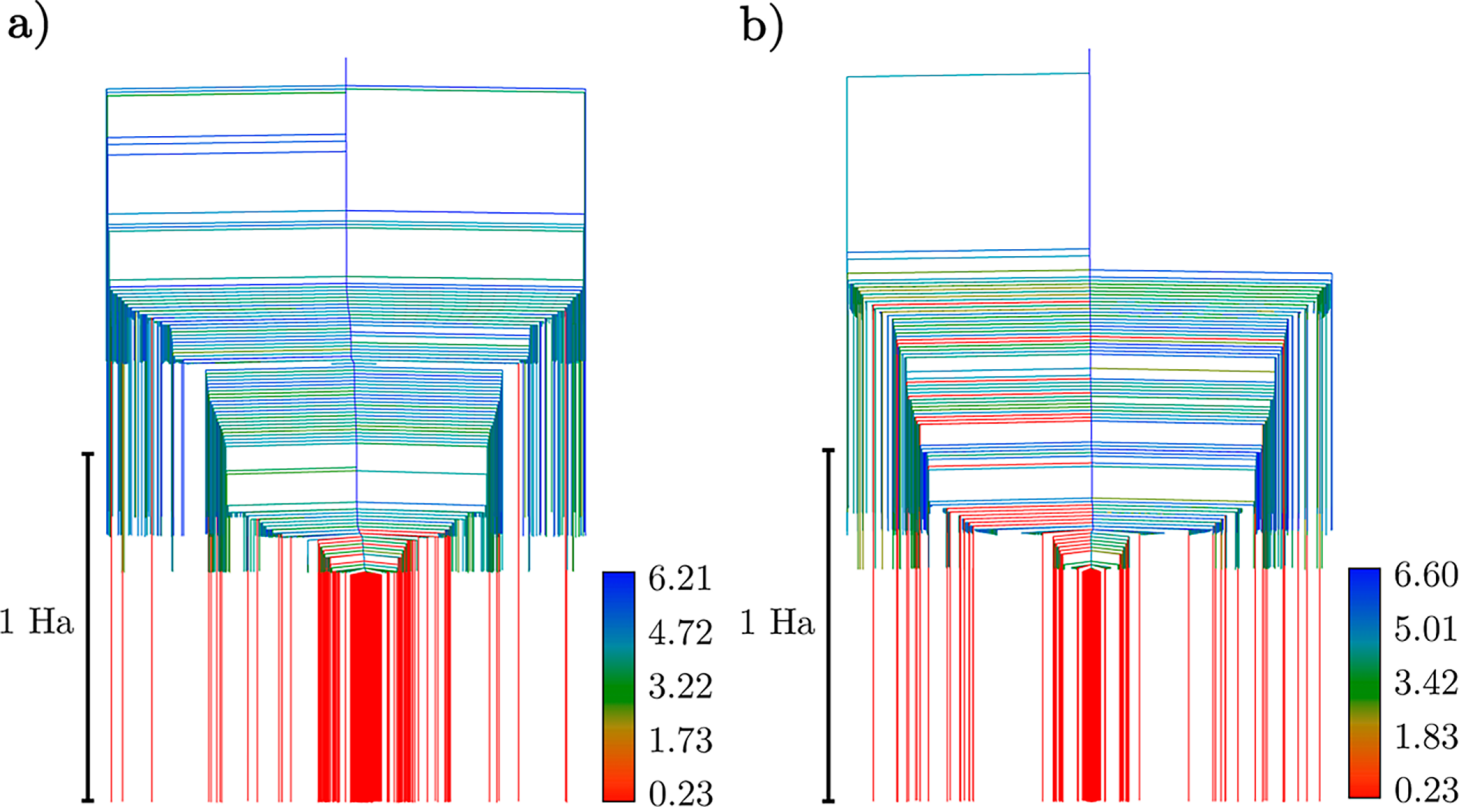
Disconnectivity graphs of the 38-operator N_2_ UCCSD ansatz with *r*_N–N_ = 1.1 Å for the interleaved (a) and singles-first (b) cases, respectively. Individual minima were merged if they are within 10^–5^ Ha and are colored by their corresponding *D* values.

**Figure 8:**
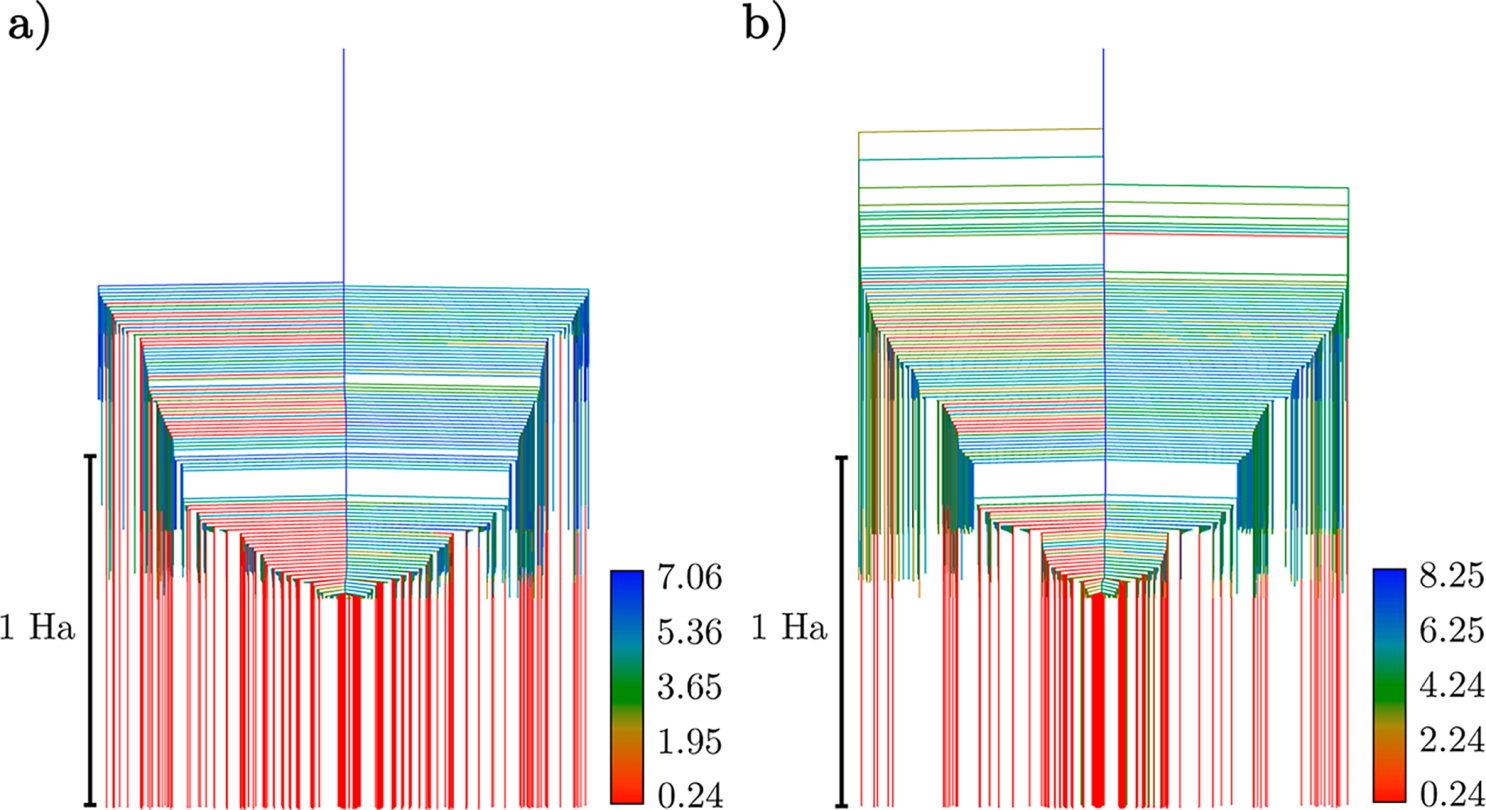
Disconnectivity graphs of the 53-operator N_2_ UCCSD ansatz with *r*_N–N_ = 1.1 Å for the interleaved (a) and singles-first (b) cases, respectively. Individual minima were merged if they are within 10^–5^ Ha and are colored by their corresponding *D* values.

As a second example, we consider the energy landscape of the UCCSD ansatz for N_2_ at *r*_N–N_ = 1.1 Å consisting of 53 parameters, of which only two are single excitations. Setting a threshold of 0.01 on the amplitude reduces the ansatz to 38 parameters, which includes the two single excitation operators. A more stringent threshold of 0.05 further decreases the number of parameters to 17, all of which correspond to double excitation operators. Disconnectivity graphs for the 38- and 53-parameter ansätze are given in Figures 7 and 8, respectively. We note that for N_2_ even in the interleaved case, there are multiple low-lying minima close to the global minimum (see Table 2 for its energy), and the spread of the minima in the lowest band exceeds chemical accuracy (see Table 2 for more details). As in the case of LiH, all these minima are characterized by low values of *D*.

**Table 2:** Value of the Lowest Energy Minimum, the Number of Sampled Connected Minima, and the Energy Difference within the Lowest Minima Neighborhood Found with Different UCCSD Ansätze for the N_2_ Molecule with *r*_N–N_ = 1.1 Å

parameters	order	*M*	Δ*E*_*l*_ (Ha)	global minimum (Ha)	FCI (Ha)
17		13	2.60 × 10^–3^	–107.552125	–107.653827
38	interleaved	46,599	4.24 × 10^–3^	–107.579747	
38	singles-first	47,369	4.26 × 10^–3^	–107.579738	
53	interleaved	62,896	1.02 × 10^–2^	–107.614436	
53	singles-first	62,911	1.02 × 10^–2^	–107.614439	

**Figure 9:**
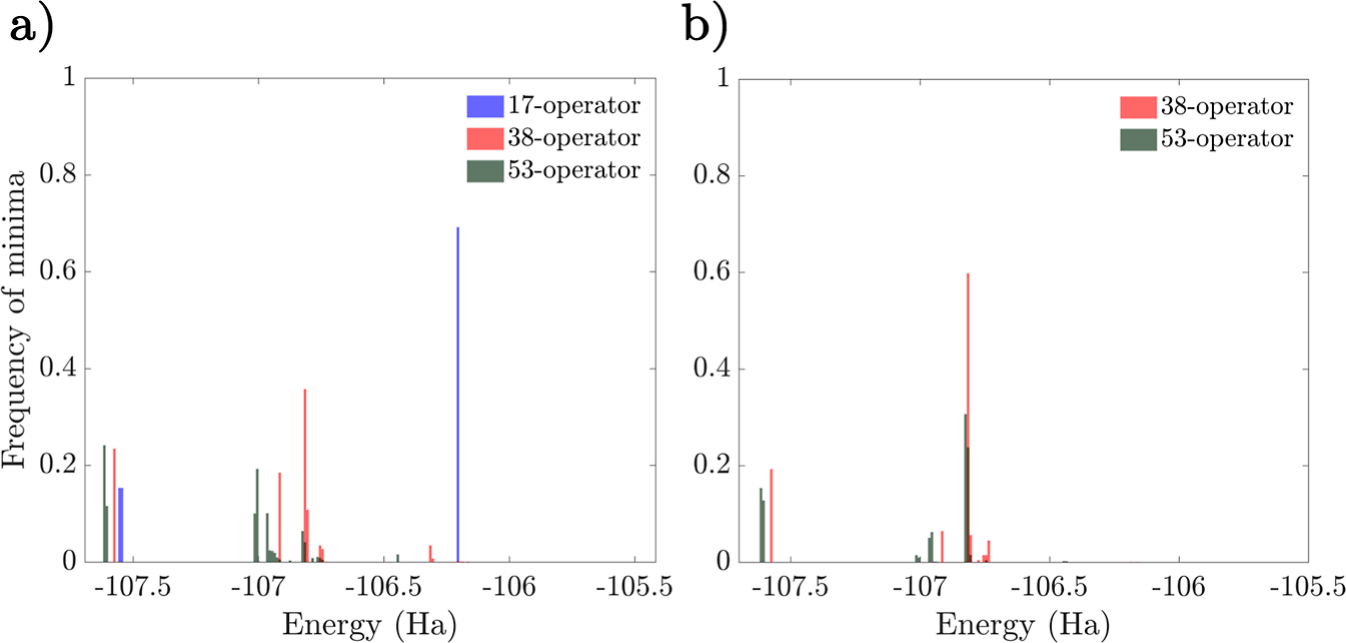
Minima histogram distributions of the interleaved (a) and singles-first (b) N_2_ UCCSD ansatz for *r*_N–N_ = 1.1 Å. The 17-, 38-, and 53-operator cases are shown in blue, red, and green, respectively.

In this case, with only one pair of spin-flipped single excitation operators, there is a less significant difference between the energy landscapes of the interleaved and singles-first expansions. Nevertheless, we can see from the energy distributions in Figure 9 that the relative number of high-energy minima is larger in the singles-first case. We also note that while restricting the ansatz to only 17 parameters has a relatively small, although important, effect on the global minimum, it significantly increases the energy of the higher minima.

**Figure 10:**
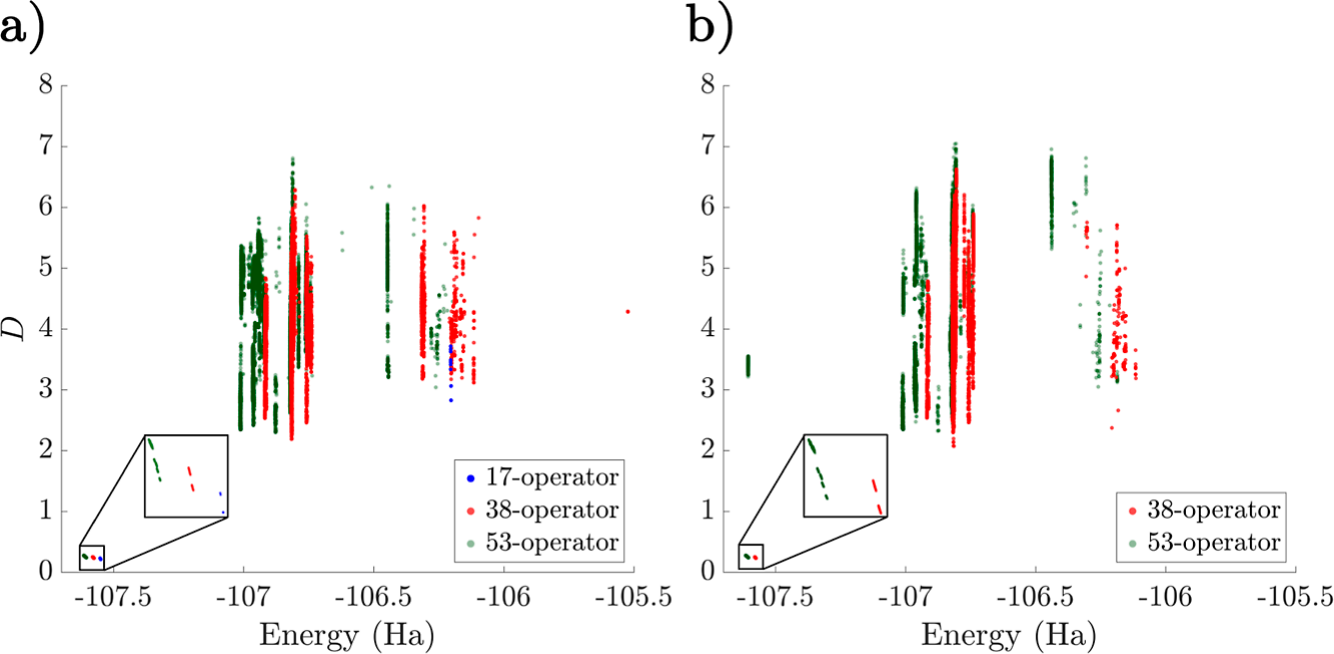
Scatter plots of the interleaved (a) and singles-first (b) N_2_ UCCSD ansatz for *r*_N–N_ = 1.1 Å based on the energy and *D* values of the minima. The 17-, 38-, and 53-operator cases are shown in blue, red, and green, respectively.

We also plot the *D* values of minima with respect to the energy for the N_2_ ansätze series (Figure 10). Once again, in the interleaved ansatz, the global minimum neighborhood corresponds to minima with low *D* values. While not as pronounced as in the LiH case, the singles-first ansatz once again introduces some low energy, high *D* minima. We note here that the low *D* value of the global minimum in the interleaved case is not merely a consequence of the molecular geometry being close to equilibrium. We expect that with increasing multideterminant character as the bond length increases, the magnitude of the UCCSD parameters (relative to the inactive angles 0 and ±π) will increase, leading to larger values of *D*. This effect is indeed observed, with the average *D* for the lowest band of minima increasing from 0.27 at *r*_N–N_ = 1.1 Å to 0.81 at *r*_N–N_ = 1.6 Å. Nevertheless, this value is significantly lower than the *D* values of minima in higher energy bands, as shown in Figure 10. These values also increase with bond length, with an average *D* of 6.03 for the stretched geometry.

**Figure 11:**
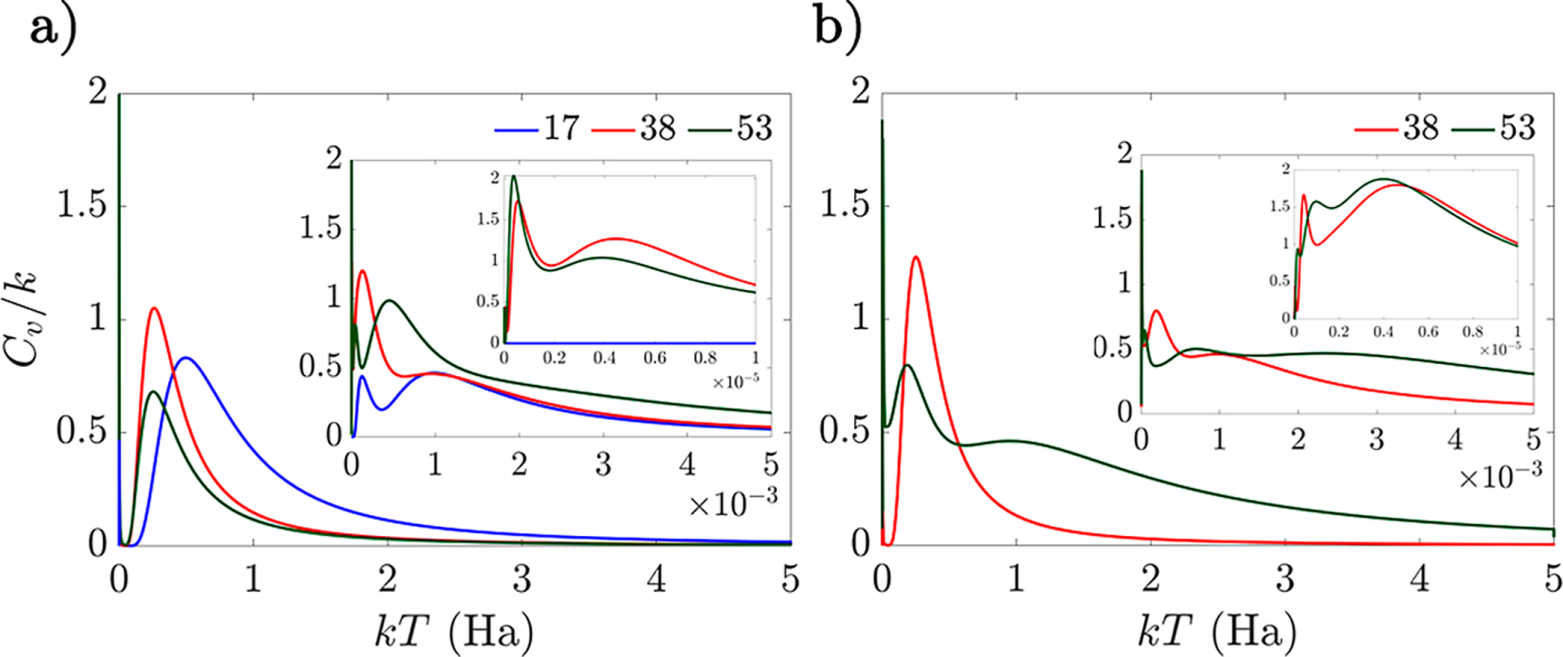
Heat capacity curves for the interleaved (a) and singles-first (b) N_2_ UCCSD ansatz for *r*_N–N_ = 1.1 Å. The 17-, 38-, and 53-operator cases for both operator orders are shown in blue, red, and green, respectively.

Finally, we compared the heat capacity curves with different numbers of operators and operator orders for the N_2_ series (Figure 11). The area under by the main peaks for the interleaved and singles-first cases generally decreases as the number of operators increases, attributable to the overall shift in minima energies in the higher energy neighborhoods toward the global minimum. However, in the case of the singles-first ansatz, the area under the main peak composed from the partial overlap of two peaks is much greater for the 53-operator case. This result arises from the more complex transitions between the slightly greater proportion of minima found in the lowest energy band, in contrast to the interleaved case, where the proportion of minima is relatively constant. As observed in the singles-first case of LiH, both operator orders of N_2_ gave rise to sharper secondary heat capacity peaks for the 53-operator case, arising from the increased energy difference between higher energy bands of minima.

## 4 Conclusions

In this contribution, we have presented energy landscapes of UCC ansätze to approximate the ground-state energies for LiH and N_2_. We compared the landscape of the full UCCSD parametrizations to truncated versions based on initial Monte Carlo approximations, as well as two alternative operator orderings: the interleaved form suggested by Evangelista et al.^33^ and the intuitive singles-first approach.

In general, we find that organization of the UCC landscapes resembles previous results for glassy systems,^65−69^ with many low-lying minima separated by large energy barriers, unlike the more funnelled energy landscapes observed for hardware-efficient and QAOA ansätze in VQAs.^17,18^ From a practical perspective, this structure suggests that landscape exploration will be relatively difficult, particularly if we are to locate the true global minimum. It is also noteworthy that the energy difference between minima in the low energy band lies within the range associated with chemical accuracy for LiH, but this is not the case for N_2_, and we anticipate that this energy range will increase with the complexity of the system.

UCC energy landscapes with truncated parametrizations are generally less complex, but the corresponding global minima lie above the result for the full UCCSD wavefunction. Nevertheless, this is a promising observation for methods like ADAPT-VQE,^9^ which attempt to construct simpler ansätze based on UCC operators. However, these formulations have been found to reach a plateau at energies above the global minimum,^15^ suggesting that the landscape remains challenging and difficult to navigate.

We find that operator ordering has a significant effect on landscape complexity, with the singles-first-order introducing many additional minima compared to the interleaved approach. This organization is shown to be due to increased flexibility in how UCC ansätze approach the ground state, allowing the optimization scheme to take large steps away from the target wave function before recovering accuracy through a later operator.

QAOA energy landscapes were generally found to be more funnelled than the present UCC landscapes, and the funnelling organization increased with the number of variational circuit layers.^17,18^ This effect might be emulated in the UCC ansatz by increasing the order of the Trotter expansion of the overall UCC operator. Thus, the methodology presented in the present work suggests further possibilities for exploiting the energy landscape approach to study different VQE ansätze used in the electronic structure problem, such as ADAPT-VQE^9^ or the recent tiled unitary product state ansatz.^16^ Another possible avenue for exploration is the study of landscapes utilizing alternative cost functions that can be used to optimize parameters in VQE, such as the variance of the energy commonly used in variational Monte Carlo methods.^81−84^ Closely related quantities are also employed in excited state algorithms,^85^ for example, in the folded-spectrum VQE approach.^86^

## Appendix A

### Jordan-Wigner transformation

The Fermionic annihilation(creation) operator *p̂* may be written as

25

where *X*, *Y*, and *Z* are the standard Pauli matrices, with *Z*_*q*_ denoting the Pauli matrix *Z* acting on qubit *q*, and σ_*p*_^±^ = *X*_*p*_ ± *iY*_*p*_. This formulation gives the following expressions for single and double excitation operators

26

27
